# Carotid artery calcification at the initiation of hemodialysis is a risk factor for cardiovascular events in patients with end-stage renal disease: a cohort study

**DOI:** 10.1186/1471-2369-12-56

**Published:** 2011-10-15

**Authors:** Masaru Nakayama, Yoriko Ura, Masaharu Nagata, Yasushi Okada, Yoko Sumida, Kanako Nishida, Hirofumi Ikeda, Yoshiki Kaizu

**Affiliations:** 1 Division of Nephrology and Clinical Research Institute, Department of Internal Medicine, National Kyushu Medical Center Hospital, 1-8-1 Jigyohama, Chuo-ku, Fukuoka 810-8563, Japan; 2 Department of Medicine and Clinical Science, Graduate School of Medical Science, Kyushu University, 3-1-1, Maidashi, Higashi-ku, Fukuoka 812-8582, Japan; 3 Department of Cerebrovascular Diseases, Cerebrovascular Center and Clinical Research Institute, National Kyushu Medical Center Hospital, 1-8-1 Jigyohama, Chuo-ku, Fukuoka 810-8563, Japan

## Abstract

**Background:**

Vascular calcification has been recognized as a risk factor for cardiovascular (CV) events in patients with end-stage renal disease (ESRD). However, the association of carotid artery calcification (CAAC) with CV events remains unknown. The aim of this study was to elucidate whether CAAC is associated with composite CV events in ESRD patients.

**Methods:**

One-hundred thirty-three patients who had been started on hemodialysis between 2004 and 2008 were included in this retrospective cohort study. These patients received multi-detector computed tomography to assess CAAC at the initiation of hemodialysis. Composite CV events, including ischemic heart disease, heart failure, cerebrovascular diseases, and CV deaths after the initiation of hemodialysis, were examined in each patient.

**Results:**

CAAC was found in 94 patients (71%). At the end of follow-up, composite CV events were seen in 47 patients: ischemic heart disease in 20, heart failure in 8, cerebrovascular disease in 12, and CV deaths in 7. The incidence of CAAC was 87% in patients with CV events, which was significantly higher than the rate (62%) in those without. Kaplan-Meier analysis showed a significant increase in composite CV events in patients with CAAC compared with those without CAAC (p = 0.001, log-rank test). Univariate analysis using a Cox hazards model showed that age, smoking, common carotid artery intima-media thickness and CAAC were risk factors for composite CV events. In multivariate analysis, only CAAC was a significant risk factor for composite CV events (hazard ratio, 2.85; 95% confidence interval, 1.18-8.00; p = 0.02).

**Conclusions:**

CAAC is an independent risk factor for CV events in ESRD patients. The assessment of CAAC at the initiation of hemodialysis is useful for predicting the prognosis.

## Background

The annual mortality rate due to cardiovascular (CV) disease in patients with end-stage renal disease (ESRD) is more than an order of magnitude greater than that in the population with normal kidney function [1]. Vascular calcification is a common complication of ESRD. Arterial disease in patients with ESRD is characterized by a high degree of intimal as well as medial calcification. Arterial intimal calcification is associated with atherosclerotic burden, resulting in arterial stenosis or occlusion; on the other hand, arterial medial calcification is related to arteriosclerosis, which causes arterial stiffening with higher systolic blood pressure, increased pulse pressure and early return of wave reflections, leading to altered coronary perfusion and left ventricular hypertrophy [2]. It has been demonstrated that vascular calcification is closely associated with an increased risk of CV events and mortality in patients with ESRD [3-9]. Therefore, the fact that CV disease is the leading cause of death in ESRD patients appears to be due in part to the presence of excess vascular calcification.

Most studies that have described the predictive value of vascular calcification for CV morbidity and mortality have focused on the following arterial sites: the coronary artery [5,8], the abdominal aorta [7,9], combined sites such as the carotid artery, abdominal aorta, iliofemoral axis and legs [3], the iliofemoral axis [4], and both the pelvis and hands [6]. On the other hand, several studies have addressed the association of carotid artery lesions with CV events and mortality in patients with ESRD. One study demonstrated that carotid artery plaque was a significant predictor of CV events [10], and another showed the relationship between carotid artery stiffening, and all-cause and CV mortality [11]. In addition, it was demonstrated that increased intima-media thickness (IMT) of the carotid artery is a significant predictor of CV deaths [12,13]. In contrast, in a population-based cohort, the presence of carotid artery calcification (CAAC), detected by ultrasonography as calcified plaque, was an independent predictor of combined vascular events such as stroke, myocardial infarction or vascular death [14]. However, to date, the specific impact of CAAC on CV morbidity and mortality has never been established in chronic kidney disease (CKD) patients, including patients with ESRD. We have previously reported the risk factors for CAAC, as evaluated by multi-detector computed tomography (MDCT), in incident hemodialysis patients by a cross-sectional study [15]. The aim of the present study was to elucidate whether the presence of CAAC at the initiation of hemodialysis could be associated with CV events after the initiation of hemodialysis.

## Methods

One-hundred thirty-five Japanese patients who were admitted to our hospital to start maintenance hemodialysis between March 2004 and December 2008 underwent carotid MDCT examination to assess CAAC. All of the patients provided written informed consent to the protocol, which was approved by the Ethics Committee of National Kyushu Medical Center Hospital. We previously reported the risk factors for CAAC and carotid atherosclerosis in these 135 patients at the initiation of hemodialysis by a cross-sectional study [15]. After discharge, all patients underwent maintenance hemodialysis at 40 other clinics. All of these clinics agreed to record the details of CV events or death after the initiation of hemodialysis. Two of 135 patients withdrew informed consent, and the remaining 133 were enrolled in this retrospective cohort study. In the present study, CV events were defined as development of the following conditions after the initiation of hemodialysis: ischemic heart disease, heart failure, cerebrovascular disease such as brain infarction and hemorrhage, and CV deaths. CV deaths were defined as death caused by ischemic heart disease, heart failure, stroke, sudden death, rupture of an abdominal aortic aneurysm, ischemic colitis, and peripheral artery disease. The date of CV events, or date and cause of death were obtained by reviewing the hospital records. During the follow-up, one patient underwent renal transplantation, and was censored at that time. When multiple events occurred in the same patient, only the time to the first event was analyzed and this first event was defined as an end-point of composite CV events. The mean follow-up period was 48.5 ± 19.0 (SD) months.

All of the enrolled patients were interviewed and underwent a clinical examination at the initiation of hemodialysis. Their medical history and outpatient records were also evaluated in detail. Demographic information (age and gender), comorbidities (ischemic heart disease, cerebrovascular disease, and peripheral artery disease), and atherosclerotic risk factors (hypertension, history of smoking, dyslipidemia, and diabetes mellitus) at the initiation of hemodialysis were recorded for each patient. Cigarette smoking was evaluated as current or past. Dyslipidemia was defined as plasma triglyceride > 150 mg/dL, plasma low-density lipoprotein cholesterol > 140 mg/dL, plasma high-density lipoprotein cholesterol < 40 mg/dL, or use of lipid-lowering drugs based on a history of dyslipidemia. In cases in which calcium carbonate, vitamin D3, erythropoiesis-stimulating agents (epoetin alfa or beta), statins, angiotensin-converting enzyme inhibitors, or angiotensin II receptor blockers had been prescribed, these prescriptions were reviewed before starting hemodialysis. Blood and urine samples were obtained for serological tests within a few days before starting maintenance hemodialysis. The systolic and diastolic blood pressures were recorded as the mean average of pre- and post-dialysis blood pressures at the last three hemodialysis sessions during hospitalization at our hospital for the initiation of hemodialysis (6 measurements). Body mass index (BMI) was calculated as the dry weight in kilograms divided by height in meters squared. The criteria for previous CV disease included a history of ischemic heart disease (documented myocardial infarction, coronary angioplasty or bypass surgery, significant coronary artery stenosis confirmed by coronary angiography, angina pectoris, or local asynergy on echocardiography and perfusion defect on dipyridamole thallium stress scintigraphy), cerebrovascular disease (documented stroke or transient ischemic attack, or lacunar infarcts verified by CT or magnetic resonance imaging), and peripheral artery disease (arteriosclerosis obliterans).

**Figure 1:**
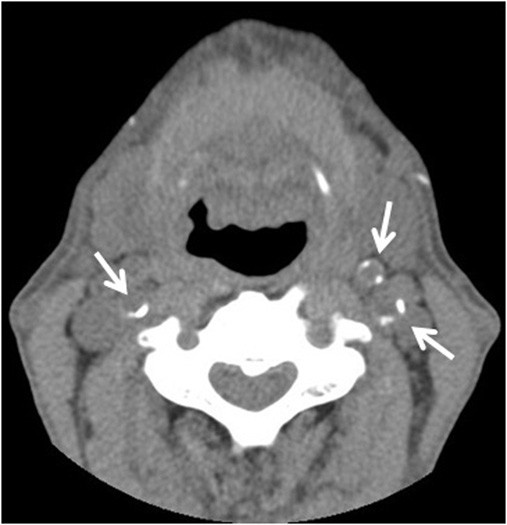
**Carotid artery calcification detected by multi-detector computed tomography at the initiation of hemodialysis was seen at the origin of the bilateral internal carotid arteries and left external carotid artery (arrows)**.

Carotid CT images were performed to detect calcification of common, internal or external carotid arteries, using a 16-row multidetector CT scanner (Multispeed Ultra 16x; GE Medical Systems, Milwaukee, WI) with 22.9 mm/s table speed, 512 × 512 pixel matrix, 0.625-mm slice thickness, 0.6-mm reconstruction index, 120 kV peak tube energy and autoregulation of tube currents (Figure 1). The presence or absence of CAAC was assessed by radiologists who were blinded to the clinical information. High-resolution B-mode ultrasound was used to assess common carotid artery (CCA)-IMT and carotid artery plaque. The presence or absence of aortic arch calcification on a plain chest X-ray was evaluated, and all subjects were assessed independently by two investigators (S.S. and K.K.) blinded to the clinical information. The CCA-IMT was taken as the distance between the lumen-intima interface and the media-adventitia interface on the B-mode ultrasound image at 3-cm before the carotid bifurcation. The mean CCA-IMT was determined for each patient and the average of both CCA-IMTs was calculated. Carotid artery plaque was defined as an area of focal increase in CCA-IMT (> 1.1 mm) and/or calcified deposits in carotid arteries. A hyperechoic structure in the walls of common, internal, or external carotid arteries with an acoustic shadow was defined as plaque calcification [16]. The ankle-brachial index (ABI) was also examined in 130 patients (98%). Those with ABI < 0.9 in either leg were assigned to the low ABI group [17]. Detailed descriptions of the methods of ultrasonography and ABI have been reported previously [15]. The MDCT, ultrasonography and ABI examinations were performed within 1 week before or after the initiation of hemodialysis.

### Statistical analysis

Continuous data are expressed as either the means ± SD or median (interquartile range) depending on their distribution, and categorical data are expressed as numbers (with %). Differences in the prevalence were evaluated using the chi-squared test and Fisher's exact test for groups containing less than five individuals in any given cell. The statistical significance of differences between groups was examined using the Wilcoxon rank sum test for nonparametric data or unpaired Student's *t*-test for parametric data. Survival curves were estimated by the Kaplan-Meier method and evaluated by log-rank test. Risk factors for outcomes were first examined using a univariate Cox proportional hazards model, and variables with a significant association (p < 0.10) were then applied to a multivariate Cox proportional hazards model. Data were statistically analyzed using the JMP8 statistical package (SAS Institute, Cary, NC, USA). A *P *value below 0.05 indicated a statistically significant difference.

## Results

### Characteristics of the patients

The mean age at the initiation of hemodialysis of the 133 patients (77 men and 56 women) in this study was 64.6 ± 10.5 years (range, 42-88). The primary causes of renal disease were chronic glomerulonephritis in 23 (17%) patients, diabetic nephropathy in 59 (44%), hypertensive nephrosclerosis in 21 (16%), amyloidosis in 9 (7%), polycystic kidney disease in 5 (4%), other defined causes in 7 (5%) and unknown in 9 (7%). The primary causes of renal diseases were diagnosed by renal biopsy in six patients, while the causes in other patients were determined based on medical history and clinical findings. Clinical characteristics at the initiation of hemodialysis of the 133 patients with and without CAAC are summarized in Table 1. The age of patients with CAAC was significantly higher than that in those without. The prevalence of hypertension, dyslipidemia and previous CV disease was significantly increased in patients with CAAC compared with those without. Systolic blood pressure and pulse pressure were higher in patients with CAAC than in those without. The value of hemoglobin was significantly lower in patients with CAAC than in those without CAAC. Calcium-phosphorus product in patients with CAAC tended to be higher compared with that in those without, but no significant difference was observed. In contrast, the value of C-reactive protein was significantly higher in patients with CAAC than in those without. As for carotid artery lesions, both the value of CCA-IMT and prevalence of carotid artery plaque were significantly higher in patients with CAAC than in those without. Half of patients with CAAC showed calcified plaque confirmed by ultrasonography, while no patients without CAAC had calcified plaque. MDCT and ultrasonography showed an agreement of 64% for the detection of carotid calcification. Aortic arch calcification was found in 54% of our subjects. The prevalence of aortic arch calcification in patients with CAAC was significantly higher than in patients without CAAC. In addition, the prevalence of low ABI was higher in patients with CAAC than in those without.

**Table 1:** Characteristics of the 133 patients with ESRD with and without CAAC at the initiation of hemodialysis

Variables	CAAC (+)	CAAC (-)	p value
No. of patients	94 (71)	39 (29)	
Age (y)	67.2 ± 10.1	58.3 ± 8.8	< 0.01
Male	57 (61)	20 (51)	0.32
Hypertension	93 (99)	32 (82)	< 0.01
Diabetes mellitus	58 (62)	18 (46)	0.10
Dyslipidemia	77 (82)	25 (64)	0.03
Smoking	45 (48)	14 (36)	0.20
Previous CVD	48 (51)	4 (10)	< 0.01
Calcium carbonate	26 (28)	13 (33)	0.52
Vitamin D3	14 (15)	5 (13)	0.75
Dose of ESA (U/month)	3000 (0-3000)	1500 (0-3000)	0.47
Statins	26 (28)	5 (13)	0.05
ACEI and/or ARB	50 (53)	20 (51)	0.84
SBP (mm Hg)	158 ± 20	150 ± 23	0.04
DBP (mm Hg)	79 ± 10	82 ± 11	0.13
Pulse pressure (mm Hg)	79 ± 16	68 ± 15	< 0.01
Body mass index (kg/m^2^)	20 (18-22)	21 (19-23)	0.04
Blood urea nitrogen (mg/dL)	92 (78-106)	88 (78-120)	0.76
Serum creatinine (mg/dL)	9.0 (7.6-10.2)	9.6 (8.3-11.2)	0.07
Up/Ucr	4.7 (2.3-8.0)	3.7 (1.4-7.3)	0.10
Serum albumin (g/dL)	3.1 ± 0.6	3.4 ± 0.6	0.06
Hemoglobin (g/dL)	8.1 ± 1.1	8.6 ± 1.5	0.02
Ca-P product (mg^2^/dL^2^)	53.6 (44.2-64.8)	51.9 (42.8-61.4)	0.44
C-reactive protein (mg/dL)	0.15 (0.05-0.44)	0.06 (0.02-0.21)	0.01
intact-PTH (pg/mL)	227 (150-315)	260 (170-420)	0.08
CCA-IMT (mm)	0.83 (0.72-1.10)	0.71 (0.57-0.80)	< 0.01
CAP	77 (82)	9 (23)	< 0.01
Calcified plaque	46 (49)	0 (0)	< 0.01
AAC	67 (71)	5 (13)	< 0.01
Low ABI	20 (22)	1 (3)	< 0.01

Values are expressed as the mean ± SD, number (%), or median (interquartile range). Abbreviations: ESRD, end-stage renal disease; CAAC, carotid artery calcification; CVD, cardiovascular disease; ESA, erythropoiesis-stimulating agents; ACEI, angiotensin-converting enzyme inhibitors; ARB, angiotensin II receptor blockers; SBP, systolic blood pressure; DBP, diastolic blood pressure; Up/Ucr, urinary protein-creatinine ratio; Ca-P, calcium-phosphorus; PTH, parathyroid hormone; CCA-IMT, common carotid artery-intima-media thickness; CAP carotid artery plaque; AAC, aortic arch calcification; ABI, ankle-brachial index.

### Comparison of clinical findings of the patients with and without CV events

The number of CV events occurring subsequent to initiation of hemodialysis was 47. Of the initial CV events, 20 were ischemic heart disease, 8 were heart failure, 12 were cerebrovascular disease, and 7 were CV deaths. Clinical characteristics at the initiation of hemodialysis of 133 patients with and without CV events are shown in Table 2. There was no difference in demographics and traditional CV risk factors between the two groups with and without CV events. The prevalence of previous CV disease did not differ between the two groups. With regard to medications which were administered after the initiation of hemodialysis, there was no difference in doses of erythropoiesis-stimulating agents or the number of patients with angiotensin-converting enzyme inhibitors and/or angiotensin II receptor blockers between the two groups, while the number of patients treated with statins was higher in patients with CV events than in those without CV events. In addition, the values of non-traditional CV risk factors, e.g., body mass index, hemoglobin, proteinuria, serum albumin, calcium-phosphorus product, C-reactive protein and intact parathyroid hormone did not show a significant difference between the two groups. Both the value of CCA-IMT and the prevalence of carotid artery plaque in patients with CV events tended to be higher compared with those in patients without, but the difference did not reach significance, and there was no difference in the prevalence of aortic arch calcification between the two groups. However, the prevalence of CAAC by MDCT or calcified plaque by ultrasonography in the former group was significantly higher than that in the latter. In addition, among the 87 patients without calcified plaque by ultrasonography, 48 patients had CAAC by MDCT and 39 patients showed no CAAC. Seventeen (35%) of the patients with CAAC had CV events and 6 (15%) of the patients without CAAC experienced CV events; the prevalence of CV events in the former was significantly higher than that in the latter (p = 0.03).

**Table 2:** Characteristics at the initiation of hemodialysis of the 133 patients with and without cardiovascular events

Variables	CV events (+)	CV events (-)	p value
No. of patients	47 (35)	86 (65)	
Age (y)	66.7 ± 9.4	63.4 ± 10.9	0.10
Male	29 (62)	48 (56)	0.51
Hypertension	46 (98)	79 (92)	0.13
Diabetes mellitus	30 (64)	46 (53)	0.25
Dyslipidemia	38 (81)	64 (74)	0.40
Smoking	26 (55)	33 (38)	0.06
Previous CV disease	21 (45)	31 (36)	0.33
Dose of ESA (U/week)	9000 (4500-9000)	9000 (9000-9000)	0.34
Statins	16 (34)	14 (16)	0.02
ACEI and/or ARB	24 (51)	41 (48)	0.71
SBP (mm Hg)	155 ± 15	156 ± 23	0.92
DBP (mm Hg)	78 ± 10	80 ± 10	0.34
Pulse pressure (mm Hg)	76 ± 12	75 ± 19	0.51
Body mass index (kg/m^2^)	20 (18-23)	21 (19-22)	0.43
Blood urea nitrogen (mg/dL)	92 (73-114)	90 (80-110)	0.60
Serum creatinine (mg/dL)	8.4 (6.9-9.7)	9.5 (8.3-10.9)	< 0.01
Hemoglobin (g/dL)	8.4 ± 1.1	8.1 ± 1.3	0.22
Up/Ucr	4.4 (2.0-7.2)	4.6 (1.8-8.1)	0.75
Serum albumin (g/dL)	3.1 ± 0.6	3.2 ± 0.6	0.43
Ca-P product (mg^2^/dL^2^)	51.5 (43.7-61.4)	53.3 (43.5-65.0)	0.58
C-reactive protein (mg/dL)	0.14 (0.05-0.44)	0.09 (0.04-0.28)	0.30
intact-PTH (pg/mL)	235 (150-302)	245 (160-381)	0.41
CCA-IMT (mm)	0.83 (0.73-0.97)	0.77 (0.64-0.95)	0.09
CAP	35 (74)	51 (59)	0.08
Calcified plaque	24 (51)	22 (26)	< 0.01
CAAC	41 (87)	53 (62)	< 0.01
AAC	28 (60)	44 (51)	0.35
Low ABI	10 (22)	11 (13)	0.18
Duration of follow-up (months)	19.2 (9.1-32.5)	51.6 (38.4-65.7)	< 0.01

Values are expressed as the mean ± SD, number (%), or median (interquartile range). Abbreviations: CV, cardiovascular; ESA, erythropoiesis-stimulating agents; ACEI, angiotensin-converting enzyme inhibitors; ARB, angiotensin II receptor blockers; SBP, systolic blood pressure; DBP, diastolic blood pressure; Up/Ucr, urinary protein-creatinine ratio; Ca-P, calcium-phosphorus; PTH, parathyroid hormone; CCA-IMT, common carotid artery-intima-media thickness; CAP carotid artery plaque; CAAC, carotid artery calcification; AAC, aortic arch calcification; ABI, ankle-brachial index.

### Risk factors for CV events

**Figure 2:**
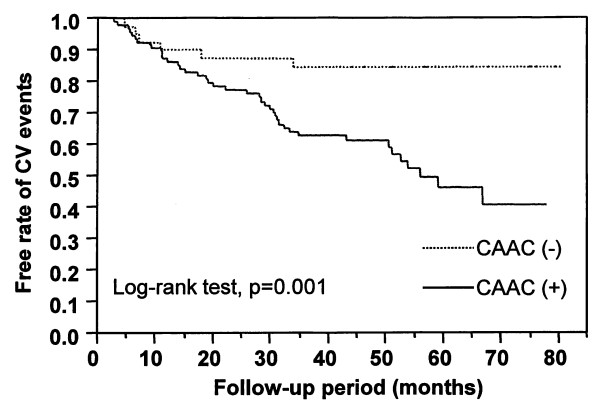
**Kaplan-Meier analysis of the free rate of CV events of 133 patients with end-stage renal disease according to the presence or absence of CAAC**. A comparison between the survival curves was significant (p = 0.001, Log-rank test). Abbreviations: CV, cardiovascular; CAAC, carotid artery calcification.

The incidence of subsequent CV events in patients with CAAC was significantly higher than that in patients without (Log-rank test, p = 0.001), as shown in Figure 2. Table 3 shows the risk factors for CV events, which were assessed by a Cox proportional hazards model. Univariate analysis showed that age, smoking, CCA-IMT, and CAAC were significant risk factors for CV events. However, other traditional CV risk factors, such as hypertension, diabetes and dyslipidemia, were not identified as risk factors. Additionally, previous CV disease was not associated with the subsequent CV events. Non-traditional CV risk factors, e.g., calcium-phosphorus product, serum albumin and C-reactive protein, were also not found to be risk factors. Furthermore, carotid artery plaque or aortic arch calcification was not associated with CV events. In the multivariate analysis, only CAAC was determined to be an independent risk factor for CV events.

**Table 3:** Risk factors for cardiovascular events by Cox analysis

	Univariate			Multivariate^a^		
	
Variable	HR	95%CI	p value	HR	95% CI	p value
Age	1.03	1.00-1.06	0.04	1.01	0.98-1.05	0.38
Male	1.27	0.71-2.32	0.43	-	-	-
Hypertension	3.27	0.71-57.9	0.15	-	-	-
Diabetes Mellitus	1.49	0.83-2.76	0.19	-	-	-
Dyslipidemia	1.35	0.68-2.97	0.41	-	-	-
Smoking	1.95	1.09-3.52	0.02	1.73	0.94-3.20	0.08
Previous CV disease	1.40	0.78-2.48	0.26	-	-	-
Pulse pressure (mm Hg)	1.00	0.99-1.02	0.83	-	-	-
Body mass index (kg/m^2^)	0.98	0.90-1.07	0.69	-	-	-
Up/Ucr	1.02	0.97-1.07	0.38	-	-	-
Serum albumin (g/dL)	0.74	0.47-1.17	0.20	-	-	-
Ca-P product (mg^2^/dL^2^)	0.98	0.96-1.00	0.11	-	-	-
C-reactive protein (mg/dL)	1.19	0.66-1.91	0.53	-	-	-
intact-PTH (pg/mL)	1.00	0.996-1.00	0.14	-	-	-
CCA-IMT (mm)	2.97	1.20-6.52	0.02	1.51	0.52-3.79	0.43
CAP	1.69	0.90-3.39	0.10	-	-	-
CAAC	3.78	1.72-9.97	< 0.01	2.85	1.18-8.00	0.02
AAC	1.52	0.85-2.77	0.15			
Low ABI	1.77	0.83-3.43	0.13	-	-	-

Abbreviations: HR, hazard ratio; CI, confidence interval; CV, cardiovascular; Up/Ucr, urinary protein-creatinine ratio; Ca-P, calcium-phosphorus; PTH, parathyroid hormone; CCA-IMT, common carotid artery-intima-media thickness; CAP, carotid artery plaque; CAAC, carotid artery calcification; AAC, aortic arch calcification; ABI, ankle-brachial index. ^a ^The multivariate model includes variables for which p < 0.10 by univariate analysis.

### Comparison of clinical findings of the patients with and without all-cause death

At the end of follow-up, 42 total deaths were recorded. The survival rates at years 3 and 5 were 78% and 66%, respectively. The causes of death were as follows: CV death in 17 patients, malignancy in 9, infection in 6, other defined causes in 5 and unknown in 5. Table 4 shows the comparison of clinical findings at the initiation of hemodialysis of 133 patients according to the presence or absence of all-cause death. Among the demographics and traditional CV risk factors, the age in patients with all-cause death was higher than that in patients without. With regard to medications which were administered after the initiation of hemodialysis, no significant difference was observed in doses of erythropoiesis-stimulating agents or the number of patients with statins or angiotensin-converting enzyme inhibitors and/or angiotensin II receptor blockers between the two groups. Diastolic blood pressure in patients with all-cause death was significantly lower than that in patients without. With regard to non-traditional CV risk factors, the C-reactive protein level was higher in patients with all-cause death than in those without. As for carotid artery lesions, both the value of CCA-IMT and prevalence of CAAC by MDCT or calcified plaque by ultrasonography in patients with all-cause death were significantly higher than in those without. The prevalence of aortic arch calcification in patients with all-cause death was higher than in those without all-cause death. In addition, the prevalence of low ABI in patients with all-cause death was significantly higher than that in patients without.

**Table 4:** Characteristics at the initiation of hemodialysis of the 133 patients with and without all-cause death

Variables	Death (+)	Death (-)	p value
No. of patients	42 (32)	91 (68)	
Age (y)	71.4 ± 8.3	61.4 ± 10.0	< 0.01
Male	27 (64)	50 (55)	0.31
Hypertension	40 (95)	85 (93)	0.67
Diabetes mellitus	23 (55)	53 (58)	0.71
Dyslipidemia	30 (71)	72 (79)	0.34
Smoking	16 (38)	43 (47)	0.32
Previous CV disease	20 (48)	32 (35)	0.17
Dose of ESA (U/week)	9000 (6000-9000)	9000 (6000-9000)	0.92
Satins	10 (24)	20 (22)	0.81
ACEI and/or ARB	16 (38)	49 (54)	0.09
SBP (mm Hg)	153 ± 15	156 ± 23	0.49
DBP (mm Hg)	77 ± 10	81 ± 10	0.03
Pulse pressure (mm Hg)	77 ± 12	75 ± 18	0.55
Body mass index (kg/m^2^)	20 (17-23)	21 (19-22)	0.23
Blood urea nitrogen (mg/dL)	89 (73-102)	92 (81-114)	0.09
Serum creatinine (mg/dL)	9.3 (6.9-10.3)	9.2 (8.0-10.7)	0.30
Up/Ucr	4.3 (1.9-7.9)	4.7 (1.9-7.9)	0.66
Serum albumin (g/dL)	3.1 ± 0.5	3.3 ± 0.6	0.11
Hemoglobin (g/dL)	8.3 ± 1.3	8.2 ± 1.3	0.86
Ca-P product (mg^2^/dL^2^)	51.2 (42.1-57.5)	53.7 (44.2-65.3)	0.18
C-reactive protein (mg/dL)	0.18 (0.07-0.51)	0.07 (0.04-0.25)	0.04
intact-PTH (pg/mL)	235 (168-273)	250 (148-390)	0.32
CCA-IMT (mm)	0.83 (0.74-1.12)	0.76 (0.64-0.90)	< 0.01
CAP	32 (76)	54 (59)	0.05
Calcified plaque	23 (55)	23 (25)	< 0.01
CAAC	37 (88)	57 (63)	< 0.01
AAC	28 (67)	44 (48)	< 0.05
Low ABI	12 (29)	9 (10)	< 0.01
Duration of follow-up (months)	29.1 (17.8-48.5)	53.6 (45.4-67.8)	< 0.01

Values are expressed as the mean ± SD, number (%), or median (interquartile range). Abbreviations: CV, cardiovascular; ESA, erythropoiesis-stimulating agents; ACEI, angiotensin-converting enzyme inhibitors; ARB, angiotensin II receptor blockers; SBP, systolic blood pressure; DBP, diastolic blood pressure; Up/Ucr, urinary protein-creatinine ratio; Ca-P, calcium-phosphorus; PTH, parathyroid hormone; CCA-IMT, common carotid artery intima-media thickness; CAP carotid artery plaque; CAAC, carotid artery calcification; AAC, aortic arch calcification; ABI, ankle-brachial index.

### Risk factors for all-cause mortality

Table 5 shows the results of the Cox proportional hazards model used to determine the risk factors for all-cause mortality. The variables with p < 0.10 by univariate analysis were as follows: age, serum albumin, C-reactive protein, CCA-IMT, carotid artery plaque, CAAC, aortic arch calcification and low ABI. When these variables were entered into the multivariate analysis, only age was identified as a risk factor for all-cause mortality; CAAC did not reach the level of statistical significance.

**Table 5:** Risk factors for all-cause mortality by Cox analysis

	Univariate			Multivariate^a^		
	
Variable	HR	95%CI	p value	HR	95% CI	p value
Age	1.08	1.05-1.12	< 0.01	1.09	1.05-1.14	< 0.01
Male	1.33	0.72-2.56	0.37	-	-	-
Hypertension	1.33	0.41-8.1 6	0.68	-	-	-
Diabetes Mellitus	0.92	0.50-1.71	0.79	-	-	-
Dyslipidemia	0.73	0.38-1.49	0.37	-	-	-
Smoking	0.81	0.43-1.49	0.50	-	-	-
Previous CV disease	1.39	0.75-2.55	0.29	-	-	-
Pulse pressure (mm Hg)	1.00	0.99-1.02	0.65	-	-	-
Body mass index (kg/m^2^)	0.96	0.87-1.05	0.33	-	-	-
Up/Ucr	1.02	0.97-1.07	0.38	-	-	-
Serum albumin (g/dL)	0.62	0.38-1.00	0.05	0.89	0.51-1.58	0.67
Ca-P product (mg^2^/dL^2^)	0.98	0.96-1.00	0.11	-	-	-
C-reactive protein (mg/dL)	1.64	0.95-2.58	0.07	1.68	0.91-2.92	0.10
intact-PTH (pg/mL)	1.00	0.996-1.00	0.14	-	-	-
CCA-IMT (mm)	3.76	1.58-8.05	< 0.01	2.20	0.76-6.00	0.14
CAP	1.80	0.92-3.86	0.09	0.59	0.24-1.61	0.29
CAAC	3.87	1.66-11.3	< 0.01	1.83	0.55-6.63	0.33
AAC	1.87	1.00-3.66	< 0.05	0.89	0.43-1.92	0.76
Low ABI	2.70	1.33-5.15	< 0.01	1.83	0.78-3.94	0.16

Abbreviations: HR, hazard ratio; CI, confidence interval; CV, cardiovascular; Up/Ucr, urinary protein-creatinine ratio; Ca-P, calcium-phosphorus; PTH, parathyroid hormone; CCA-IMT, common carotid artery-intima-media thickness; CAP, carotid artery plaque; CAAC, carotid artery calcification; AAC, aortic arch calcification; ABI, ankle-brachial index. ^a ^The multivariate model includes variables for which p < 0.10 by univariate analysis.

## Discussion

The principal finding of the present cohort study was that CAAC at the initiation of hemodialysis was confirmed to be an independent risk factor for subsequent CV events in patients with ESRD. Most of the previous studies assessing vascular calcification in ESRD have been conducted in patients on maintenance dialysis. In contrast, to date, only two studies by Block et al. have been conducted in incident dialysis patients [8,18]. One of those studies demonstrated that the coronary artery calcification score at the initiation of hemodialysis is a significant predictor for all-cause mortality [8]. In the parent study, subjects without coronary artery calcification at the time of hemodialysis initiation did not accrue substantial calcification during the first 18 months of dialysis independent of phosphate binder treatment [18]. The current study evaluated CAAC only at the initiation of hemodialysis, and did not re-examine CAAC after initiating hemodialysis. Further studies will thus be needed to elucidate whether or not the progression of CAAC differs between patients with and without CAAC at the initiation of hemodialysis.

Most previous studies have used ultrasonography to evaluate CAAC as calcified plaque, while in the present study CAAC was assessed using MDCT. Carotid ultrasonography cannot provide a detailed visualization of vascular calcification due to acoustic shadows; however, CT scans can provide such a visualization [19]. Furthermore, it has been reported that MDCT can precisely determine the mineral content of non-moving *ex vivo *human carotid endarterectomy specimens [20]. Therefore, it is considered that MDCT is a sensitive method for the detection of CAAC. However, the method does not allow differentiation of medial from intimal calcification. It has been reported that obvious intimal hyperplasia and striking medial calcification were observed in an analysis of the morphology of large elastic arteries in ESRD patients [21], and another study uncovered heavily calcified atherosclerotic plaques in patients with ESRD [22]. Considering these facts, intimal and medial calcification might coexist in affected vessels in ESRD. In our previous study, advanced age, hypertension, and disordered calcium-phosphorus metabolism were shown to be independent risk factors for CAAC [15], and these risk factors are considered to be associated with intimal and/or medial calcification [4,23,24]. Accordingly, CAAC as assessed by MDCT in the current study is suggested to reflect both intimal and medial calcification. Furthermore, this suggestion could explain the fact that the prevalence of CAAC measured by MDCT was twice that of calcified carotid plaque by ultrasonography in this study, thus supporting the findings of a previous report which demonstrated ultrasonography to be less accurate than unenhanced CT in quantifying carotid artery calcium [25].

On the other hand, as a method for detecting arterial calcification, simple radiographs are considered inexpensive and involve less radiation exposure than CT. In our study, aortic arch calcification on a plain X-ray was also assessed, and the prevalence was 54%, which was similar to that reported by a previous study [26]. In our study, the presence of aortic arch calcification was found not to be a risk factor for CV events; in contrast, CAAC by MDCT was confirmed to be an independent risk factor for CV events. Accordingly, although a precise comparison of the effectiveness of the two methods would seem to be difficult due to the different arterial sites (carotid artery on MDCT vs. aortic arch on X-ray), the evaluation of CAAC by MDCT might be useful for predicting subsequent CV events, which could offset the risk conferred by the radiation exposure.

It has been reported that calcification in ESRD patients occurs in the arterial intima in association with atherosclerotic plaque [22]. Furthermore, London et al. noted that intimal calcification was usually observed in older patients with a clinical history of atherosclerosis and traditional risk factors for atherosclerotic disease, and that the odds ratio for the presence of intimal calcification significantly increases in the presence of carotid artery plaque [4]. In our previous report, it was also demonstrated that CAAC was closely associated with carotid artery plaque, suggesting the association of carotid intimal rather than medial calcification with carotid atherosclerosis [15]. Based on these findings, patients with vascular calcification, including CAAC, appear to have a greater atherosclerotic burden compared to those without, and this condition might contribute to the poor prognosis in ESRD patients. In a population-based study, a positive correlation between calcification in CAAC and in coronary artery calcification evaluated by multislice CT was found [27]. It was also reported that the coronary artery calcium score is associated with an increased risk of coronary heart disease events in asymptomatic subjects [28]. Based on these findings, CAAC might be associated with the occurrence of cardiac events, although the coronary artery calcification was not assessed in our study.

Most investigators have postulated that arterial medial calcification, a common finding in dialysis patients, is related to vascular stiffness, which in turn results in adverse CV outcomes. It has been considered that arterial medial calcification likely increases arterial stiffness and ventricular afterload [2,29]. Increased arterial stiffness, in turn, has been associated with increased all-cause or CV mortality in ESRD patients [11,30,31]. Increased arterial stiffness could contribute to high pulse pressure [4]. It has also been reported that higher systolic blood pressure, lower diastolic blood pressure and wider pulse pressure all confer greater CV and all-cause mortality risk [32]. However, in the present study, there was no significant difference in the values of systolic blood pressure, diastolic blood pressure, or pulse pressure between the group with and the group without CV events, or between the group with and the group without all-cause death. In the Cox proportional hazards model, these parameters did not have predictive value for either CV events or all-cause death. Additionally, the impact of vascular calcification on CV prognosis according to whether vascular calcification existed in the intimal or medial layers has not been directly investigated in previous studies using electron-beam CT or MDCT. Accordingly, it remains unknown to what extent medial calcification could have contributed to the development of CV events or mortality in ESRD patients in our study.

Unlike in previous reports [10,12,13], carotid artery plaque or CCA-IMT was not found to be an independent risk factor for CV events or all-cause death in the current study. Given that CAAC was an independent risk factor for CV events in our study, the assessment of CAAC at the initiation of hemodialysis could have greater significance when predicting the subsequent prognosis. Disordered calcium-phosphorus metabolism is considered to be one of the modifiable risk factors for vascular calcification in CKD [33]. It has been revealed that increased serum phosphorus and calcium-phosphorus product are associated with the development of vascular calcification in uremic patients [23,24], and that higher serum phosphorus concentrations that are still within the normal range are associated with a greater prevalence of vascular calcification in predialysis patients [34]. In our previous report, calcium-phosphorus product was a significant risk factor for the development of CAAC at the initiation of hemodialysis [15]. In addition, several studies have identified hyperphosphatemia as an independent risk factor for CV morbidity and mortality in dialysis patients [35,36], and it was also demonstrated that in predialysis patients elevated serum phosphate levels were independently associated with increased all-cause mortality [37]. Therefore, the control of disordered calcium-phosphorus metabolism, especially hyperphosphatemia, is considered to be one of the key roles for the attenuation of vascular calcification or prolonged survival in predialysis or dialysis patients; however, few studies have demonstrated the benefits of reducing serum phosphate concentration on vascular calcification and mortality. Recent studies have demonstrated that sevelamer, a non-calcium phosphate binder, significantly reduces the progression of vascular calcification in predialysis [38], prevalent [39] and incident dialysis patients [18]. However, it remains unclear whether the effect of sevelamer on vascular calcification is due to phosphate binding without an increase in calcium load or results from the lipid-lowering effect of sevelamer. Furthermore, given that a significant difference in all-cause or CV mortality was not found in patients receiving sevelamer compared with calcium-based phosphate binders [40,41], it is still unknown whether this benefit is associated with all-cause or CV mortality.

The current study has some limitations. Our study was a retrospective cohort and the sample size was relatively small. In the present study, CAAC showed a significant risk factor for all-cause mortality in univariate analysis, but was not identified as a risk factor for all-cause mortality in multivariate analysis, which was inconsistent with the previous studies [3-5,7,8]. In the present study, the proportion of CV deaths to all-cause deaths was 40%, similar to that reported by Okuno et al. [7]; conversely, most other studies reported a proportion of 46% to 64% [3-5]. In addition to the relatively small sample size, this lower proportion of CV deaths may have prevented the identification of an independent association of CAAC with all-cause mortality. Furthermore, the result that only age was an independent risk factor for all-cause mortality in our study might be attributable to relatively older subjects with a mean age of 64.6 years at the initiation of hemodialysis. MDCT assessment depended on the presence or absence of CAAC, but calcium content was not scored. If the CAAC score were to be quantified, then the relationship of CAAC scores with CV events could be more precisely documented.

## Conclusions

In conclusion, it was confirmed that CAAC, as detected by MDCT at the initiation of hemodialysis, was an independent risk factor for subsequent CV events. Considering the result that carotid artery plaque or CCA-IMT, surrogate markers of subclinical atherosclerosis, did not have predictive value for CV events, it was suggested that the assessment of CAAC, among various carotid artery alterations, was more useful in predicting the prognosis in ESRD patients.

## Competing interests

The authors declare that they have no competing interests.

## Authors' contributions

MN^1 ^was involved in the study design, sample collection, analysis and interpretation of the data and in the writing of the report. YU acquired the data and participated in the interpretation of the data. MN^2 ^participated in the analysis and interpretation of the data. YO participated in the interpretation of the data. YS, KN, HI, and YK participated in the sample collection. All authors have read and approved the final version of the manuscript.

## Pre-publication history

The pre-publication history for this paper can be accessed here:

http://www.biomedcentral.com/1471-2369/12/56/prepub
